# Andrological effects of SARS-Cov-2 infection: a systematic review and meta-analysis

**DOI:** 10.1007/s40618-022-01801-x

**Published:** 2022-05-09

**Authors:** G. Corona, W. Vena, A. Pizzocaro, F. Pallotti, D. Paoli, G. Rastrelli, E. Baldi, N. Cilloni, M. Gacci, F. Semeraro, A. Salonia, S. Minhas, R. Pivonello, A. Sforza, L. Vignozzi, A. M. Isidori, A. Lenzi, M. Maggi, F. Lombardo

**Affiliations:** 1 Endocrinology Unit, Medical Department, Azienda Usl, Maggiore-Bellaria Hospital, Bologna, Italy; 2 Unit of Endocrinology, Diabetology and Medical Andrology, IRCSS, Humanitas Research Hospital, Rozzano, Milan Italy; 3 Department of Experimental Medicine, “Sapienza” University of Rome, Policlinico Umberto I Hospital, Rome, Italy; 4 Andrology, Women’s Endocrinology and Gender Incongruence Unit, Department of Experimental and Clinical Medicine, University of Florence, Florence, Italy; 5 Department of Anaesthesia, Intensive Care and EMS, Maggiore Hospital Bologna, Bologna, Italy; 6 Department of Minimally Invasive and Robotic Urologic Surgery and Kidney Transplantation, Careggi University Hospital (AOUC), University of Florence, Florence, Italy; 7 Division of Experimental Oncology/Unit of Urology, URI, IRCCS Ospedale San Raffaele, Milan, Italy; 8 University Vita-Salute San Raffaele, Milan, Italy; 9 Department of Urology, Imperial Healthcare NHS Trust, Charing Cross Hospital, London, UK; 10 Dipartimento di Medicina Clinica e Chirurgia, Sezione di Endocrinologia, Unità di Andrologia e Medicina della Riproduzione e della Sessualità Maschile e Femminile, Università Federico II di Napoli, Naples, Italy; 11 Staff of UNESCO Chair for Health Education and Sustainable Development Baldi E, Federico II University, Naples, Italy; 12 Endocrinology Unit, “Mario Serio” Department of Experimental and Clinical Biomedical Sciences, University of Florence, Florence, Italy

**Keywords:** SARS-CoV-2, COVID-19, Testosterone, Sperm, Vaccination, Hypogonadism

## Abstract

**Purpose:**

The short- and long-term andrological effects of coronavirus disease 2019 (COVID-19) have not been clarified. Our aim is to evaluate the available evidence regarding possible andrological consequences of COVID-19 either on seminal or hormonal parameters. The safety of the COVID-19 vaccines in terms of sperm quality was also investigated.

**Methods:**

All prospective and retrospective observational studies reporting information on severe acute respiratory syndrome coronavirus 2 (SARS-Cov-2) mRNA semen and male genitalia tract detection (*n* = 19), as well as those reporting data on semen analysis (*n* = 5) and hormonal parameters (*n* = 11) in infected/recovered patients without any arbitrary restriction were included.

**Results:**

Out of 204 retrieved articles, 35 were considered, including 2092 patients and 1138 controls with a mean age of 44.1 ± 12.6 years, and mean follow-up 24.3 ± 18.9 days. SARS-CoV-2 mRNA can be localized in male genitalia tracts during the acute phase of the disease. COVID-19 can result in short-term impaired sperm and T production. Available data cannot clarify long-term andrological effects. Low T observed in the acute phase of the disease is associated with an increased risk of being admitted to the Intensive Care Unit or death. The two available studies showed that the use of mRNA COVID-19 vaccines does not affect sperm quality.

**Conclusions:**

The results of our analysis clearly suggest that each patient recovering from COVID-19 should be monitored to rule out sperm and T abnormalities. The specific contribution of reduced T levels during the acute phase of the infection needs to be better clarified.

**Supplementary Information:**

The online version contains supplementary material available at 10.1007/s40618-022-01801-x.

## Introduction

The identification and rapid worldwide dissemination of the novel severe acute respiratory syndrome coronavirus 2 (SARS-CoV-2), underpinning the coronavirus disease 2019 (COVID-19), led the World Health Organization (WHO) to declare the status of a pandemic in March 2020 [1]. Since the preliminary epidemiological data has been available, a clear sex disparity has been evident, with males, although not more frequently affected, often experiencing worse outcomes when compared to women [1–3]. The reasons underlying this association are probably multifactorial and include both social and cultural factors supporting the higher prevalence of associated morbidities observed in men when compared to women [1]. In addition, the possible contribution of hormonal factors and, in particular, testosterone (T) has also been proposed [1, 2, 4, 5]. The angiotensin converting enzyme 2 (ACE2), and the transmembrane protease, serine 2 (TMPRSS2), both crucial for viral cell entry, are highly expressed in the male genital tract and both modulated by T activity [3, 6, 7]. Limited evidence has emerged regarding the presence of SARS-CoV-2 in the male genital tract, as well as in seminal fluid [7, 8]. Similarly, the reported testis discomfort experienced, at least in a subset of patients during COVID-19 acute phase, appears to support the potential development of SARS-COV-2-related epididymal-orchitis in a number of cases [7]. Similar data has previously been reported for other coronaviruses [7]. Taken together, these observations have clearly emphasized the necessity of a more systematic evaluation of COVID-19 affected men to rapidly identify acute (semen localization and orchitis) as well as possible chronic andrological complications (i.e., infertility and hypogonadism) related to the infection. In particular, the safety and potential consequences of the utilization of reproductive cells from SARS-CoV-2-positive subjects represents a critical challenge to couples and clinicians involved in assisted reproductive care. It is noteworthy that few studies have investigated the short- and long-term andrological consequences of COVID-19. Furthermore, the lack of data derived from phase III trials related to fertility safety of COVID-19 vaccines, along with the aforementioned possible andrological consequences, still represents one the main reasons for accepting vaccination in men seeking fertility treatments [9].

The aim of the present study is to systematically review and meta-analyze all available data regarding possible short- and long-term andrological effects of COVID-19. In particular, the primary outcome is the detection rate (DR) of SARS-Cov-2 mRNA in the male genital tract and semen of infected subjects. Secondary outcomes include the comparison of semen and hormonal parameters between COVID-19 patients and controls. Finally, information regarding the safety of the COVID-19 vaccines on sperm quality was investigated.

## Methods

This meta-analysis was performed in line with the Preferred Reporting Items for Systematic Reviews and Meta-Analyses (PRISMA) reporting guideline [see Supplementary file 1]. The protocol of this study is published on the website of the University of York (Centre for Reviews and Dissemination) https://www.crd.york.ac.uk/PROSPERO (CRD42021275185).

### Search strategy

Two different extensive Medline, Embase and Cochrane searches using MeSH terms were performed.

The first search was focused on the selection of all studies evaluating the presence SARS-Cov-2 mRNA in seminal fluid of infected subjects including the following keywords: (“sarscov 2”[MeSH Terms] OR “sarscov 2”[All Fields] OR “covid”[All Fields] OR “covid 19”[MeSH Terms] OR “covid 19”[All Fields]) AND (“sperm s”[All Fields] OR “spermatozoa”[MeSH Terms] OR “spermatozoa”[All Fields] OR “sperm”[All Fields] OR “sperms”[All Fields]).

The second search was mainly focused on the selection of all studies comparing hormonal and seminal sperm parameters in infected subjects compared to controls, including the following keywords: (“covid 19”[All Fields] OR “covid 19”[MeSH Terms] OR “covid 19 vaccines”[All Fields] OR "covid 19 vaccines”[MeSH Terms] OR “covid 19 serotherapy”[All Fields] OR “covid 19 serotherapy”[Supplementary Concept] OR “covid 19 nucleic acid testing”[All Fields] OR “covid 19 nucleic acid testing”[MeSH Terms] OR “covid 19 serological testing”[All Fields] OR “covid 19 serological testing”[MeSH Terms] OR “covid 19 testing”[All Fields] OR “covid 19 testing"[MeSH Terms] OR “sarscov 2”[All Fields] OR “sarscov 2”[MeSH Terms] OR “severe acute respiratory syndrome coronavirus 2”[All Fields] OR “ncov”[All Fields] OR “2019 ncov”[All Fields] OR ((“coronavirus”[MeSH Terms] OR “coronavirus”[All Fields] OR “cov”[All Fields]) AND 2019/11/01:3000/12/31[Date—Publication])) AND (“testosterone”[MeSH Terms] OR “testosterone”[All Fields] OR “testosterone”[All Fields] OR “testosterones”[All Fields] OR “testosterone s”[All Fields]).

Data from January 1 2020 up to August 31 2021 were restricted to English-language articles and studies including human participants. The identification of relevant studies was performed independently by three of the authors (F.P, W.V and A.P), and conflicts were resolved by the first investigator (G.C). All the data identified during the first analysis were checked in a second wave analysis by three of the authors (G.R, D.P and E.B). Possible further conflicts were discussed and resolved by the first investigator (G.C). We did not employ search software but hand-searched the bibliographies of retrieved papers for additional references. All the authors adequately contributed to the analysis of the paper and reviewed the final version of the manuscript. The main source of information was derived from published articles.

### Study selection

All prospective and retrospective observational studies reporting information on SARS-Cov-2 mRNA semen and male genital tract detection of COVID-19 subjects, as well as those reporting data on semen analysis and hormonal parameters in infected/recovered patients as compared with controls (when available), without any arbitrary restriction were included (see Supplementary Fig. 1 and Table 1) [4, 5, 8, 10–38]. In addition, studies comparing semen parameters before and after COVID-19 vaccination were also included in the analysis [39, 40].

No country restrictions were applied. Case reports were excluded from the analysis (see Supplementary Fig. 1). Studies not specifically reporting on at least hormonal or sperm parameters were also excluded from the analysis.

### Outcome and quality assessment

The primary outcome was the DR of SARS-Cov-2 mRNA in the male genital tract and semen of infected subjects. Secondary outcomes included the comparison of semen and hormonal parameters between COVID-19 patients and controls. The effect of several risk factors—including age, associated morbidities, time from diagnosis as well as disease severity—on DR were investigated. Similarly, the impact of disease duration after the appearance of first symptoms and type of control populations for semen and hormonal parameters were also analyzed. Finally, the possible impact of COVID-19 vaccination in healthy controls was evaluated. The quality of trials included was assessed using the Cochrane criteria [41]. In particular, we considered the following criteria: the weaknesses of the designs that have been used (such as noting their potential to ascertain causality), the execution of the studies through a careful assessment of their risk of bias, especially the potential for selection bias and confounding to which all observational studies are susceptible, and the potential for reporting biases, including selective reporting of outcomes.

### Statistical analysis

Heterogeneity in DR was assessed using I^2^ statistics. Even when low heterogeneity was detected, a random-effect model was applied because the validity of tests of heterogeneity can be limited with a small number of component studies. We used funnel plots and the Begg adjusted rank correlation test to estimate possible publication or disclosure bias [42]; however, undetected bias may still be present, because these tests have low statistical power when the number of trials is small. Overall DR is expressed as mean percentage (95% confidence interval).

In addition, a meta-regression analysis was performed to test the effect of different parameters on SARS-COV-2 mRNA DR. Following on that, potential predictors of DR were included as continuous variables: age, time from diagnosis of the disease, associated morbidities (including hypertension, diabetes mellitus), as well as disease severity. All data were calculated using Comprehensive Meta-analysis Version 2, Biostat (Englewood, NJ, USA).

## Results

### General descriptive data

Out of 93 and 111 retrieved articles, 35 were included in the study (Table 1). Among them, 31 prospectively investigated different outcomes, whereas only four retrospective data analyses had been performed in the remaining studies (Table 1). The study flow is summarized in Supplementary Fig. 1. The characteristics of the retrieved trials (including parameters on trial quality) are reported in Table 1 and Supplementary Tables I–II. Retrieved trials included 2092 patients and 1138 controls. Mean age of included population was 44.1 ± 12.6 years, and the mean follow-up 24.3 ± 18.9 days. Finally, two studies, including overall 88 subjects (mean age 38.6 ± 6.4 years), investigated the effect of COVID-19 vaccination on semen parameters (Table 1).

**Table 1:** Characteristics of trials included in the meta-analysis

Author	Study design	Study subjects (n°)	Controls (n°)	Mean age (years)	Days to dignosis	Mild disease (%)	Mean BMI (kg/m^2^)	Smokers (%)	HPT (%)	DM (%)	DR	Sperm outcomes	Hormonal outcomes	Vaccination outcomes
Çayan et al. 2020 [10]	P	175	46	45,8		50	24.02						X	
Guo et al. 2020 [26]	P	23		41	32	78.3					X			
Holtmann et al. 2020 [11]	P	18	14	35	47	77.8	25,28	5.4	0	0	X	X		
Kayaaslan et al. 2020 [12]	P	16		33.5	2.3	68.7					X			
Li et al. 2020* [13]	P	23	22	40.8	25,8	39.1					X	X		
Li et al. 2020* [8]	P	38		31.6	10.6				2.6		X			
Ning et al. 2020 [38]	R	17		35	27	100					X			
Pan et al. 2020 [14]	P	34		37	31	100	25		9		X			
Pavone et al. 2020 [15]	P	9		37.8	42.2		27.2	55.6	22.2		X			
Rastrelli et al. 2020 [4]	P	31		65		78		3.7	51.7	29.6			X	
Rawlings et al. 2020 [16]	P	6		38	12				0	0	X			
Salciccia et al. 2020 [17]	P	20	9	66.2		31		55.1	51.7	30.9			X	
Song et al. 2020 [18]	P	13		33	29.5	15.4		0			X			
Yang et al. 2020 [19]	R	12		65	42	0			33.3		X			
Achua et al. 2021 [20]	R	6		56	11	0			50	33.3	X			
Burke et al. 2021 [21]	P	18		32	6	15.8					X			
Camici et al. 2021 [22]	P	24	24	50.5	9	59.3			12.5	8.3			X	
Cinislioglu et al. 2021 [23]	P	358	92	65.3		42.7	26	73.1	54	21.7			X	
Dhindsa et al. 2021 [24]	P	66	24	64.5		26.7	27.58						X	
Gacci et al. 2021 [25]	P	38		49.4			26.64	81.6				X		
Gonzalez et al. 2021[39]	P	45		37.1										X
Kadihasanoglu et al. 2021 [27]	P	89	143	49.9		31.2								X
Lanser et al. 2021[28]	P	155		66	18		26.4		46.5	35.7				X
Ma et al. 2021 [29] **	P	12		33.2	78.5	8.3					X			
Ma et al. 2021 [29] **	P	119	273	39									X	
Machado et al. 2021 [30]	P	15		23.2	4.2						X			
Maleki et al. 2021 [31]	P	84	105	34.2		28.6	25.62					X		
Okçelik et al. 2021 [32]	P	23	19	35.5									X	
Ruan et al. 2021 [33]	P	70	145	30.8	15	14.9	24.45	25,6	0	0	x	X		
Safrai et al. 2021 [40]	R	43		32.55										X
Salonia et al. 2021 [5]	P	286	281	52		71.2	26.46		25.5				X	
Saylam et al. 2021 [34]	P	30		35.6	1						X			
Sharma et al. 2021 [35]	P	11		29.7	40.9	81.8					X			
Temiz et al. 2021 [36]	P	10	10	36.8			26.56	21,6	0	3	x	x	X	
Xu et al. 2021 [37]	P	39	22	60.7		51.3	25.74		41	15.4			x	

P = prospective; R = retrospective; HPT = arterial hypertension; DM = diabetes mellitus; *different study; **same study, different cohort

### Male genital tract SARS-Cov-2 mRNA detection rate

**Fig. 1:**
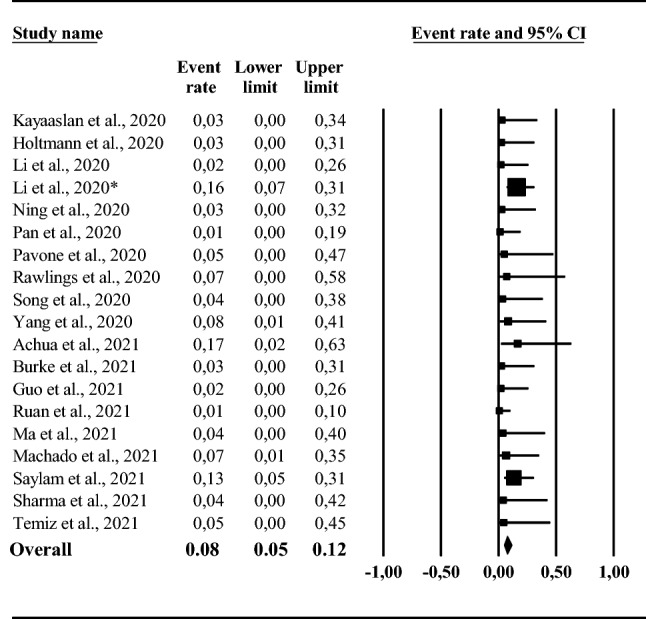
Male genitalia tract mRNA SARS-Cov-2 detection rate (%)

Among the retrieved trials, 19 studies analyzed the possible presence of SARS-Cov-2 mRNA in male genital tract (DR) samples. Among the available studies, 17 investigated the possible presence of SARS-Cov-2 mRNA directly in the semen, whereas two evaluated viral presence from testis autopsies of deceased subjects due to COVID-19 (see Supplementary Table 1). The *I*^2^ was 0; *p* = 0.665. Mean crude DR was 8 [5; 12]% (Fig. 1). A funnel plot and Begg adjusted rank correlation test (Kendall’s τ: 0.09; *p* = 0.576) suggested no major publication bias (Supplementary Fig. 2). Similar data were found when retrospective studies were compared to prospective ones (DR = 9 [6; 14]% vs. 2 [1; 7]%, respectively; *Q* = 0.05, *p* = 0.82) or after the exclusion of those studies evaluating the presence of SARS-Cov-2 in testis autopsy (7 [5; 11]%). Conversely, the DR was significantly lower when low quality studies were excluded from the analysis (DR = 8 [5; 12]% vs. 9 [3; 26]%, respectively; *Q* = 5,27, *p* = 0.02).

**Fig. 2:**
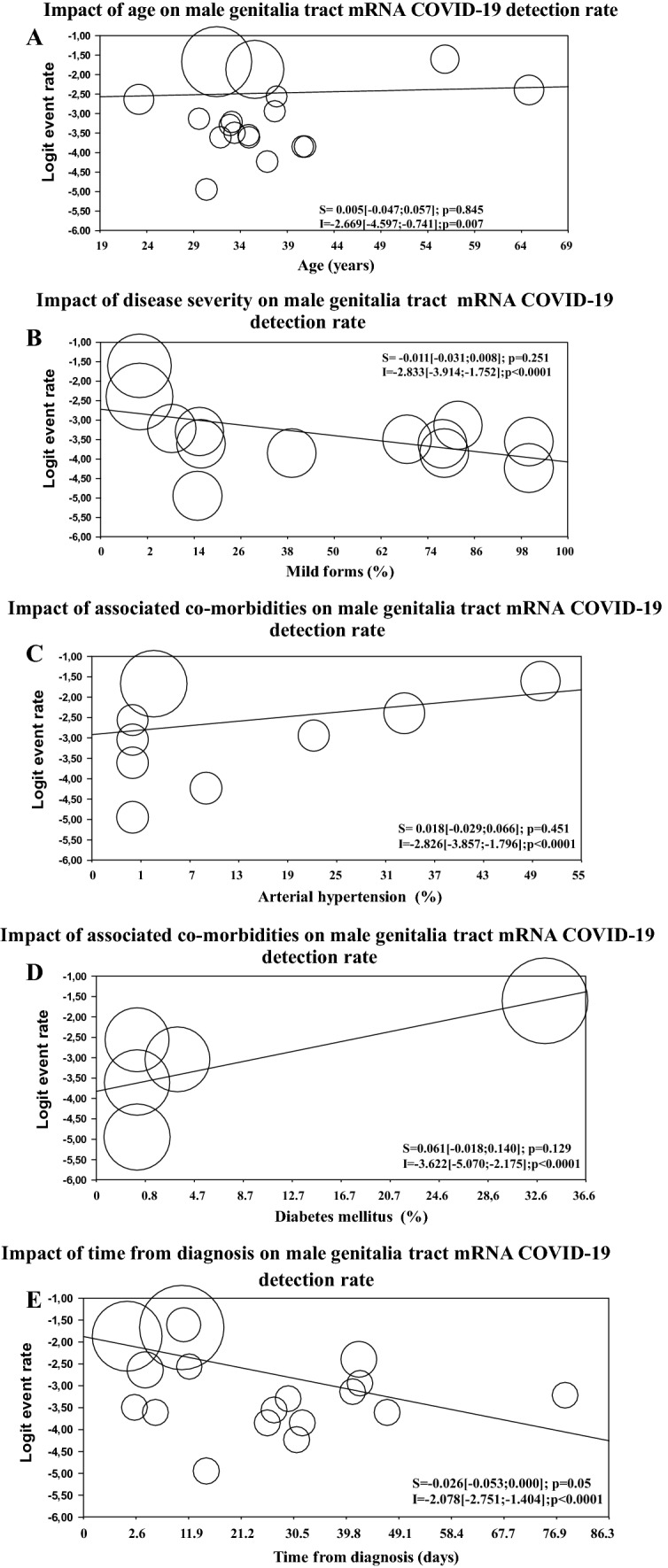
Influence of age (**A**), disease severity (**B**), arterial hypertension (**C**), diabetes mellitus (**D**), and time from diagnosis (**E**) on male genitalia tract mRNA COVID-19 detection rate. The size of the circles indicates sample dimension

Meta-regression analysis showed that DR was not influenced by patient age or by disease severity or associated morbidities (Fig. 2A–D). Conversely, DR was significantly and inversely related to COVID-19 diagnosis timing (Fig. 2E). Accordingly, the DR was significantly higher in those studies assessing the viral mRNA presence in the semen less than 11 days after the diagnosis (*Q* = 5.611; *p* = 0.018; see also Supplementary Fig. 3, panel A). The latter was confirmed even when those studies evaluating the presence of SARS-Cov-2 in testis autopsy were excluded from the analysis (Q = 5.951; *p* = 0.015, Panel B).

### Semen parameters

**Fig. 3:**
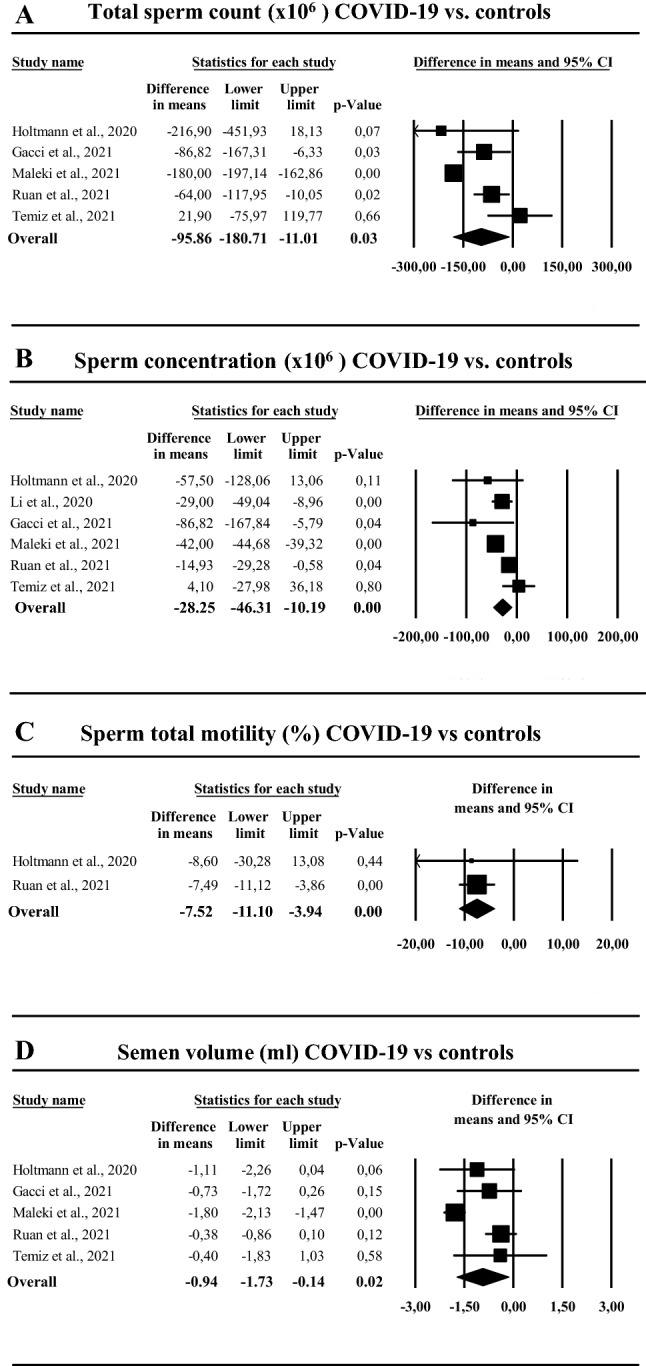
Semen parameters in COVID-19 subjects as compared to controls: total sperm count (**A**), sperm concentration (**B**), sperm total motility (**C**), semen volume (**D**)

Five trials evaluated the effect of COVID-19 on semen parameters, compared to healthy controls. In addition, one study compared the same data between hospitalized and not hospitalized infected patients (Supplementary Table 1). When all studies were considered, COVID-19 was associated with a significant reduction of total sperm count, sperm concentration and total sperm motility (Fig. 3A–C), whereas no difference in sperm morphology or progressive motility was observed (not shown). In addition, a lower seminal volume in men with COVID-19 was also detected, when compared to controls (Fig. 3D). Similar results were observed when the only study not considering healthy controls [25] was excluded from the analysis (Supplementary Fig. 4 Panels A–C). Finally, the exclusion of those studies considering subjects with positive oropharyngeal swab [11, 13, 36] from the analysis only partially attenuated the observed negative results (Supplementary Fig. 5, Panels A–C).

### Hormonal parameters

Among the included studies, 11 trials evaluated the effect of COVID-19 on different hormonal parameters, including FSH (*n* = 8), LH (*n* = 9) and total testosterone (*n* = 11) (See also Table 1 and Supplementary Tables I–II). In addition, four and five studies investigated, at baseline, the relationship between low total T level and worse COVID-19 outcomes, including probability of being admitted to ICU and mortality risk, respectively (See also Supplementary Table 1).

**Fig. 4:**
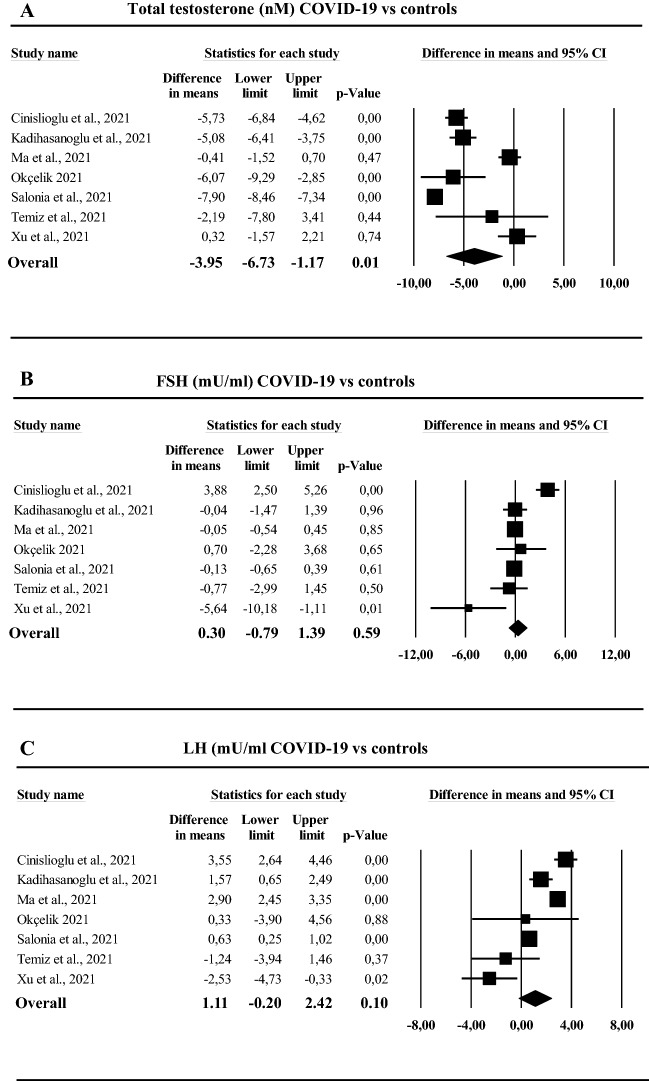
Hormonal parameters in COVID-19 subjects as compared to controls: total testosterone (**A**), follicular stimulating hormone (FSH; **B**), luteinizing hormone (LH; **C**)

SARS-Cov-2-infected patients were characterized by reduced total T levels, whereas no difference in either LH or FSH levels was observed (Fig. 4A–C). Similar results were obtained when patients with severe diseases were compared to those with milder forms (Supplementary Fig. 6 Panels A–C).

By performing sensitivity analyses, the negative effects of SARS-Cov-2 infection on T levels were confirmed when only those studies that included patients in the acute phase were investigated (mean difference in total T levels − 2.19 [− 7.08; − 1.20] nmol/l; *p* = 0.01) but not in the only study [36] including subjects in the recovering phase (mean difference in total T levels − 2.19 [− 7.80; 3.41] nmol/l; *p* = 0.44) (See also supplementary Fig. 7 Panel A). Similar to what was observed for seminal parameters, the exclusion of those studies not considering healthy controls only partially attenuated the results (see also Supplementary Fig. 7, Panel B). Similar data were observed considering LH and FSH levels (not shown).

**Fig. 5:**
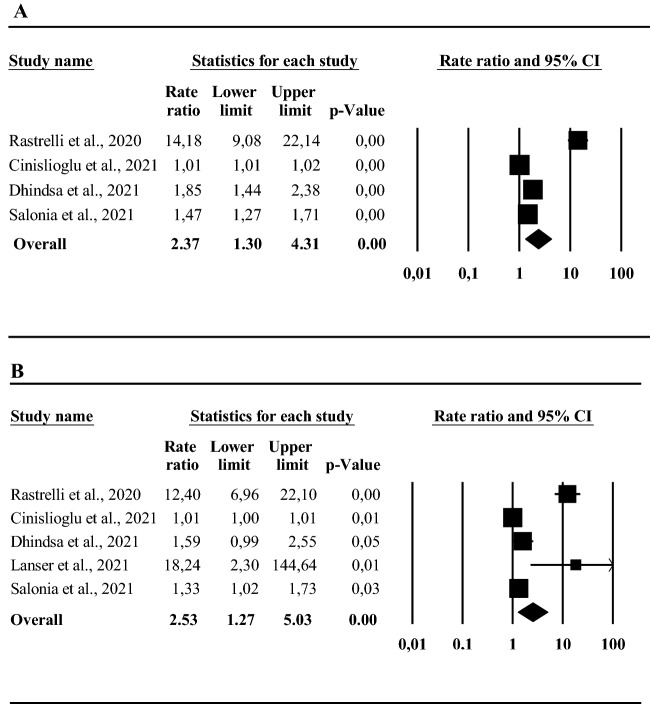
Fully adjusted risk to be admitted to Intensive Care Unit (**A**) or to die (**B**) due to COVID-19 according to low baseline T levels

Finally, when the effects of reduced T levels at baseline on COVID-19 outcomes were investigated, low T resulted in up to four- and fivefold increased risk to be admitted to the Intensive Care Unit (ICU) or to die, after the adjustment for confounders (Fig. 5A and B).

### Vaccination and semen parameters

Two studies evaluated the possible impact of COVID-19 vaccination on semen parameters in healthy volunteers. Among the subjects included, the vast majority received BNT162b2 mRNA Covid-19 vaccine whereas a small fraction received mRNA-1273 COVID-19 vaccine (see Supplementary Table 1). No significant effect of vaccination in sperm motility and semen volume was observed (Supplementary Fig. 8, Panels B and C), whereas a positive effect on sperm concentration was detected (Supplementary Fig. 8, Panel A). No further data for analysis of other semen parameters were available.

## Discussion

Our data suggest that SARS-CoV-2 mRNA can be detected in the semen only in the acute phase of COVID-19 infection but data are not conclusive. The possible testicular localization of the virus can result in impaired sperm and T production. Low T observed in the acute phase of the disease is intimately associated with an increased risk of worsening outcomes. Finally, the use of mRNA COVID-19 vaccines does not affect semen parameters.

Paoli et al. [43] did not find SARS-CoV-2 mRNA in seminal fluid and urine in 31-year-old men in the recovered phase of the infection. The same authors confirmed this result in two mild COVID-19 patients in the acute phase with a positive nasopharyngeal swab and in recovered patients with a negative swab [44]. Similar results were reported by Pan et al. [14]. The Italian Society of Andrology and Sexual Medicine (SIAMS), based on the available evidence, produced a specific *Position Statement* not supporting the presence of SARS-COV-2 in semen and the risk of sexual transmission [6]. Following these observations, several authors have further investigated the presence of SARS-CoV-2 in semen but to date only two studies documented semen viral mRNA detection in a relevant number of patients [8, 34]. Li et al. [8] in May 2020 reported for the first time the detection of SARS-CoV-2 in the semen in a limited number of patients after a relative short interval from the onset of COVID-19 symptoms. The latter study was presumably conducted in patients with a severe form and during the acute phase of the disease. This condition may correspond to a higher blood viral load and, thus, a higher chance of reaching other organs and body fluids including semen; at the same time, a confined environment with severe cases of viral disease is more likely to be susceptible to contamination. Likewise, the study of Saylam et al. [34] found four patients with SARS-CoV-2-positive semen during the viremia period of the disease. However, they were recovered in an intensive care unit and, despite a positive swab, little evidence was provided for the presence of viremia. The presence of fever and the severity of the infection have been proposed as possible facilitatory mechanisms for detection of the virus in the semen [7]. Our data indicates that the stage of the disease is the only positive predicting factor. It can be speculated that the seminal identification of the virus in the early phase of the infection can be the consequence of the alteration of Blood–Testis Barrier or secondary to its excretion in the seminal fluid. The majority of available studies provide limited information regarding the method of semen collection and preparation. Hence, the possibility that SARS-CoV-2 semen localization could reflect possible contamination from the feces, urine, hands or respiratory droplets cannot be excluded. Accordingly, DR was higher in low quality studies when compared to higher quality reports. In line with the latter hypothesis, a recent study, applying a new RT-PCR, specifically validated to detect SARS-CoV-2 RNA in sperm, did not document any positive test in patients evaluated from 6 to 181 days after a positive SARS-CoV-2 nasopharyngeal PCR test [45]. Similarly, SARS-CoV-2 mRNA was not found in 111 semen samples cryopreserved in the Florentine bank [46].

Sperm quality was significantly impaired in men with COVID-19 compared to controls. The latter results were essentially confirmed when only recovered subjects were considered. Several direct and indirect mechanisms have been proposed to explain this negative impact on sperm production. The ACE2 receptor has been documented on seminiferous duct cells including the spermatogonia, Leydig cells (LC) and Sertoli cells (SC). Although the specific role of the renin–angiotensin system in testicular function regulation has not been completely elucidated, ACE2 has been proposed as playing a putative role in steroidogenesis regulation. In addition, angiotensin II can impair sperm motility and fertility through type 1 and type 2 receptors (AT1 and AT2) leading to cell apoptosis and senescence.

Conversely, data on the expression of TMPRSS2 in the male genitalia tract are more conflicting [47]. The lack of co-expression of ACE2 and TMPRSS2 on sperm cells has been suggested to reduce the risk of viral direct damage [48]. However, it is important to recognize that, besides ACE2 and TMPRSS2, several other proteins have been shown the capacity to interact with SARS-CoV-2 during virus cell internalization. Among them, the human tyrosine-protein kinase receptor (AXL), a member of the TAM receptor family, highly expressed in Sertoli and Leydig cells can facility SARS-CoV-2 entrance into human pulmonary epithelial cells in an ACE2-independent manner [49]. Hence, after testis localization, SARS-CoV-2 can potentially enter into LC and SC, contributing to impaired T and sperm production. In addition, the inflammatory response due to viral localization within the testis can induce the development of an intense local immune reaction, supporting the development of a viral orchitis, potentially evolving into a vasculitis or to an autoimmune response [3, 7]. In addition, although never directly demonstrated, available evidence indicates that spermatozoa are able to bind to SARS-CoV-2 supporting a potential direct viral insult to spermatozoa [7]. Furthermore, local inflammation and an increased concentration of seminal leucocytes might increase oxidative stress, leading to sperm DNA breakage [7]. Finally, drugs used for COVID-19 treatment including antivirals, antibiotics and steroids can all negatively affect sperm as well as T production [7]. Despite this evidence, a recent study, including 120 SARS-CoV-2-infected subjects tested after a mean of 52.7 days from COVID-19 infection, showed that sperm parameters progressively improved according to the time lapse since virus infection [45]. Data derived from the same study also documented a strong inverse correlation between semen parameters and COVID-19 immune reaction whereas no correlation with fever was observed [45]. Taken together the available data indicate an immunologic rather than a fever-derived sperm damage. In addition, long-term sperm quality recovery was also reported [45]. Present data cannot adequately clarify the latter point due to limited follow-up of the included studies.

LC damage can contribute to impairment in T production, as documented in the current study. Ma et al. [29] demonstrated significantly higher serum LH levels and a lower T to LH ratio (T/LH) in COVID-19 patients compared to controls. Similarly, Rastrelli et al. [4] in a small sample of subjects with COVID-19 reported that a worsening clinical status was associated not only with reduced T levels but also with an increased serum LH concentration. Conversely, Salonia et al. [5] showed that secondary hypogonadism was more frequently observed in SARS-CoV-2-infected patients compared to controls. The development of secondary hypogonadism is a frequent complication of several acute and chronic illnesses. This might represent a protective mechanism, by switching off T-dependent functions (such as reproduction and/or physical and sexual activity) that are not desirable when the physical condition is ailing [50]. Present data seem to support the latter hypothesis. A secondary or at least a mixed hypogonadism is associated with COVID-19 during the acute phase, which does not persist in the recovery phase. However, Salonia et al. [51] in a 7-month follow-up of their data, reported that although total T levels increased over time, more than 50% of men who recovered from the disease still had circulating T levels suggestive of a condition of hypogonadism. Despite these considerations, we here report that low T observed at hospital admission was associated with worse disease outcomes. Similarly, low endogenous T has been associated with an increased risk of overall mortality as well as cardiovascular (CV) mortality and morbidity in the general population [52]. The role of low T in the stratification of CV risk is still conflicting. Several associated morbidities including metabolic diseases [53, 54], drugs [55], HIV [56] as well as heart failure, obstructive pulmonary disease, chronic kidney diseases, and bowel inflammatory diseases have been associated with reduced T levels [57]. Salonia et al. [51] showed that the higher the burden of comorbid conditions at presentation, the lower the probability of T level recovery over time after COVID-19. The real significance of T replacement therapy (TRT) on long-term outcomes in the latter conditions is still conflicting and the optimization of the disease status, as well as weight loss, in the case of obesity, or drug withdrawal when possible, can ameliorate T levels [57, 58]. Functional hypogonadism is the emerging term frequently used to describe the latter cases [57]. Preset data, however, indicated that low T is associated with an increased risk of worsening COVID-19 outcomes, even after the adjustment for possible confounders, supporting a primary pathogenetic role of hypogonadism. Accordingly, T has been demonstrated to have an anti-inflammatory effect in either preclinical [59, 60] or clinical studies [61]. Unfortunately, no data on the effect of TRT in COVID-19 are available [4]. In addition, genetic polymorphism of androgen receptors and ACE2 may modulate susceptibility to SARS-COV-2 infection and COVID-19 outcomes in males [62].

Phase III clinical trials showed that all the available COVID-19 vaccines had an acceptable efficacy/safety profile [63]. Up to January 28rd, 2022, a total of 9.854.237.363 vaccine doses had been administered worldwide (https://covid19.who.int/) with and excellent safety/efficacy profile [64]. A recent study performed in the US indicates that COVID-19 vaccines prevented more than 139,000 deaths during the first five months that they were available with an estimated economic value ranging from $625 billion and $1.4 trillion [65]. Since reproductive toxicity was not evaluated in the clinical trials, one of the reasons for vaccine hesitancy is represented by the potential negative effects on fertility. Gonzalez et al. [39] reported the first evidence of the safety of mRNA-derived COVID-19 vaccines on sperm quality in a small sample of volunteers. Similar results were reported in the same period by Safrai et al. [40]. By meta-analyzing this data, we can confirm the safety of COVID-19 vaccination on sperm parameters. It should be recognized that only mRNA-derived vaccines were used in the available studies, supporting the necessity of further evidence with the use of other types of vaccines to confirm these results.

Several limitations should be recognized. The vast majority of the meta-analyzed data are derived from observational studies, which present an important risk of bias due to the lack of completeness of follow-up and the accrual of missing data. A further limitation is that estimating reproduction numbers for SARS-CoV-2 presents challenges due to the high proportion of infections not correctly detected by health systems, due to paucity or even the lack of symptoms and to changes in testing policies, thus resulting in different proportions of infections being detected over time and between countries. Hence, the reproducibility of our data warrants caution. The detection of RT-PCR reflects only the presence of the viral RNA and does not necessarily indicate the presence of the virus. The follow-up period is limited. Hence, long-term andrological effects of COVID-19 infection should be investigated though larger and longer studies. Similarly, the impact of associated morbidities must be better evaluated. Finally, the possible use of anti-androgen drugs could have represented a further source of bias. However, no sufficient information was available in the considered studies.

In conclusion, present data show that COVID-19 is associated with short-term sperm and T impairment, whereas long-term consequences have still not been sufficiently clarified. Limited evidence indicates no effects of COVID-19 vaccination on sperm quality. Hence, each patient recovered from COVID-19 should be accurately monitored to rule out sperm and T abnormalities. Specific contribution of reduced T levels during the acute phase of the infection still needs to be better clarified.

## Supplementary Information

Below is the link to the electronic supplementary material.

Supplementary file1 (DOCX 23 KB)

Supplementary file2 (DOCX 17 KB)

Supplementary file3 (DOCX 45 KB)

Supplementary file4 (DOCX 40 KB)

Supplementary file5 (DOCX 43 KB)

Supplementary file6 (DOCX 40 KB)

Supplementary file7 (DOCX 40 KB)

Supplementary file8 (DOCX 95 KB)

Supplementary file9 (DOC 86 KB)

Supplementary file10 (DOCX 23 KB)

## References

[1] Corona G, Pizzocaro A, Vena W, Rastrelli G, Semeraro F, Isidori AM (2021). Diabetes is most important cause for mortality in COVID-19 hospitalized patients: systematic review and meta-analysis. Rev Endocr Metab Disord.

[2] Salonia A, Corona G, Giwercman A, Maggi M, Minhas S, Nappi RE (2021). SARS-CoV-2, testosterone and frailty in males (PROTEGGIMI): a multidimensional research project. Andrology.

[3] Pivonello R, Auriemma RS, Pivonello C, Isidori AM, Corona G, Colao A (2020). Sex disparities in COVID-19 severity and outcome: are men weaker or women stronger?. Neuroendocrinology.

[4] Rastrelli G, Di Stasi V, Inglese F, Beccaria M, Garuti M, Di Costanzo D (2021). Low testosterone levels predict clinical adverse outcomes in SARS-CoV-2 pneumonia patients. Andrology.

[5] Salonia A, Pontillo M, Capogrosso P, Gregori S, Tassara M, Boeri L (2021). Severely low testosterone in males with COVID-19: a case-control study. Andrology.

[6] Corona G, Baldi E, Isidori AM, Paoli D, Pallotti F, De Santis L (2020). SARS-CoV-2 infection, male fertility and sperm cryopreservation: a position statement of the Italian Society of Andrology and Sexual Medicine (SIAMS) (Società Italiana di Andrologia e Medicina della Sessualità). J Endocrinol Invest.

[7] He Y, Wang J, Ren J, Zhao Y, Chen J, Chen X (2021). Effect of COVID-19 on male reproductive system—a systematic review. Front Endocrinol (Lausanne)..

[8] Li D, Jin M, Bao P, Zhao W, Zhang S (2020). Clinical characteristics and results of semen tests among men with coronavirus disease 2019. JAMA Netw Open.

[9] Berry SD, Johnson KS, Myles L, Herndon L, Montoya A, Fashaw S (2021). Lessons learned from frontline skilled nursing facility staff regarding COVID-19 vaccine hesitancy. J Am Geriatr Soc.

[10] Çayan S, Uğuz M, Saylam B, Akbay E (2020). Effect of serum total testosterone and its relationship with other laboratory parameters on the prognosis of coronavirus disease 2019 (COVID-19) in SARS-CoV-2 infected male patients: a cohort study. Aging Male.

[11] Holtmann N, Edimiris P, Andree M, Doehmen C, Baston-Buest D, Adams O (2020). Assessment of SARS-CoV-2 in human semen-a cohort study. Fertil Steril.

[12] Kayaaslan B, Korukluoglu G, Hasanoglu I, Kalem AK, Eser F, Akinci E (2020). Investigation of SARS-CoV-2 in semen of patients in the acute stage of COVID-19 infection. Urol Int.

[13] Li H, Xiao X, Zhang J, Zafar MI, Wu C, Long Y (2020). Impaired spermatogenesis in COVID-19 patients. EClinicalMedicine..

[14] Pan F, Xiao X, Guo J, Song Y, Li H, Patel DP (2020). No evidence of severe acute respiratory syndrome-coronavirus 2 in semen of males recovering from coronavirus disease 2019. Fertil Steril.

[15] Pavone C, Giammanco GM, Baiamonte D, Pinelli M, Bonura C, Montalbano M (2020). Italian males recovering from mild COVID-19 show no evidence of SARS-CoV-2 in semen despite prolonged nasopharyngeal swab positivity. Int J Impot Res.

[16] Rawlings SA, Ignacio C, Porrachia M, Du P, Smith DM, Chaillon A (2020). No evidence of SARS-CoV-2 seminal shedding despite SARS-CoV-2 persistence in the upper respiratory tract. Open Forum Infect Dis.

[17] Salciccia S, Del Giudice F, Gentile V, Mastroianni CM, Pasculli P, Di Lascio G (2020). Interplay between male testosterone levels and the risk for subsequent invasive respiratory assistance among COVID-19 patients at hospital admission. Endocrine.

[18] Song C, Wang Y, Li W, Hu B, Chen G, Xia P (2020). Absence of 2019 novel coronavirus in semen and testes of COVID-19 patients†. Biol Reprod.

[19] Yang M, Chen S, Huang B, Zhong JM, Su H, Chen YJ (2020). Pathological findings in the testes of COVID-19 patients: clinical implications. Eur Urol Focus.

[20] Achua JK, Chu KY, Ibrahim E, Khodamoradi K, Delma KS, Iakymenko OA (2021). Histopathology and ultrastructural findings of fatal COVID-19 infections on testis. World J Mens Health.

[21] Burke CA, Skytte AB, Kasiri S, Howell D, Patel ZP, Trolice MP (2021). A cohort study of men infected with COVID-19 for presence of SARS-CoV-2 virus in their semen. J Assist Reprod Genet.

[22] Camici M, Zuppi P, Lorenzini P, Scarnecchia L, Pinnetti C, Cicalini S (2021). Role of testosterone in SARS-CoV-2 infection: a key pathogenic factor and a biomarker for severe pneumonia. Int J Infect Dis.

[23] Cinislioglu AE, Cinislioglu N, Demirdogen SO, Sam E, Akkas F, Altay MS (2021). The relationship of serum testosterone levels with the clinical course and prognosis of COVID-19 disease in male patients: a prospective study. Andrology.

[24] Dhindsa S, Zhang N, McPhaul MJ, Wu Z, Ghoshal AK, Erlich EC (2021). Association of circulating sex hormones with inflammation and disease severity in patients with COVID-19. JAMA Netw Open.

[25] Gacci M, Coppi M, Baldi E, Sebastianelli A, Zaccaro C, Morselli S (2021). Semen impairment and occurrence of SARS-CoV-2 virus in semen after recovery from COVID-19. Hum Reprod.

[26] Guo L, Zhao S, Li W, Wang Y, Li L, Jiang S (2021). Absence of SARS-CoV-2 in semen of a COVID-19 patient cohort. Andrology.

[27] Kadihasanoglu M, Aktas S, Yardimci E, Aral H, Kadioglu A (2021). SARS-CoV-2 pneumonia affects male reproductive hormone levels: a prospective. Cohort Study J Sex Med.

[28] Lanser L, Burkert FR, Thommes L, Egger A, Hoermann G, Kaser S (2021). Testosterone deficiency is a risk factor for severe COVID-19. Front Endocrinol (Lausanne)..

[29] Ma L, Xie W, Li D, Shi L, Ye G, Mao Y (2021). Evaluation of sex-related hormones and semen characteristics in reproductive-aged male COVID-19 patients. J Med Virol.

[30] Machado B, Barcelos Barra G, Scherzer N, Massey J, Dos Santos LH, Henrique Jacomo R (2021). Presence of SARS-CoV-2 RNA in semen-cohort study in the United States COVID-19 positive patients. Infect Dis Rep.

[31] HajizadehMaleki B, Tartibian B (2021). COVID-19 and male reproductive function: a prospective, longitudinal cohort study. Reproduction.

[32] Okçelik S (2021). COVID-19 pneumonia causes lower testosterone levels. Andrologia.

[33] Ruan Y, Hu B, Liu Z, Liu K, Jiang H, Li H (2021). No detection of SARS-CoV-2 from urine, expressed prostatic secretions, and semen in 74 recovered COVID-19 male patients: a perspective and urogenital evaluation. Andrology.

[34] Saylam B, Uguz M, Yarpuzlu M, Efesoy O, Akbay E, Çayan S (2021). The presence of SARS-CoV-2 virus in semen samples of patients with COVID-19 pneumonia. Andrologia.

[35] Sharma AP, Sahoo S, Goyal K, Chandna A, Kirubanandhan S, Sharma V (2021). Absence of SARS-CoV-2 infection in the semen of men recovering from COVID-19 infection: an exploratory study and review of literature. Andrologia.

[36] Temiz MZ, Dincer MM, Hacibey I, Yazar RO, Celik C, Kucuk SH (2021). Investigation of SARS-CoV-2 in semen samples and the effects of COVID-19 on male sexual health by using semen analysis and serum male hormone profile: a cross-sectional, pilot study. Andrologia.

[37] Xu H, Wang Z, Feng C, Yu W, Chen Y, Zeng X (2021). Effects of SARS-CoV-2 infection on male sex-related hormones in recovering patients. Andrology.

[38] Ning J, Li W, Ruan Y, Xia Y, Wu X, Hu K, Ding X, Wu X, Yu L, Zhou J, Mao Z, Xu W, Yu W, Cheng F. Effects of 2019 novel coronavirus on male reproductive system: a retrospective study. Preprints 2020.

[39] Gonzalez DC, Nassau DE, Khodamoradi K, Ibrahim E, Blachman-Braun R, Ory J (2021). Sperm parameters before and after COVID-19 mRNA vaccination. JAMA.

[40] Safrai M, Reubinoff B, Ben-Meir A. BNT162b2 mRNA Covid-19 vaccine does not impair sperm parameters. medRxiv. 2021:2021.04.30.21255690. 10.1016/j.rbmo.2022.01.008 PMC8801893 35279377

[41] Higgins JP, Altman DG, Gøtzsche PC, Jüni P, Moher D, Oxman AD (2011). The Cochrane Collaboration’s tool for assessing risk of bias in randomised trials. BMJ.

[42] Begg CB, Mazumdar M (1994). Operating characteristics of a rank correlation test for publication bias. Biometrics.

[43] Paoli D, Pallotti F, Colangelo S, Basilico F, Mazzuti L, Turriziani O (2020). Study of SARS-CoV-2 in semen and urine samples of a volunteer with positive naso-pharyngeal swab. J Endocrinol Invest.

[44] Paoli D, Pallotti F, Nigro G, Mazzuti L, Hirsch MN, Valli MB (2021). Molecular diagnosis of SARS-CoV-2 in seminal fluid. J Endocrinol Invest.

[45] Donders GGG, Bosmans E, Reumers J, Donders F, Jonckheere J, Salembier G (2021). Sperm quality and absence of SARS-CoV-2 RNA in semen after COVID-19 infection: a prospective, observational study and validation of the SpermCOVID test. Fertil Steril.

[46] Marchiani S, Dabizzi S, Degl'Innocenti S, Fino MG, Torcia MG, Paoli D (2022). Safety issues in semen banks during the COVID-19 pandemic: data from a European survey. J Endocrinol Invest.

[47] Borges E, Setti AS, Iaconelli A, Braga D (2021). Current status of the COVID-19 and male reproduction: a review of the literature. Andrology.

[48] Stanley KE, Thomas E, Leaver M, Wells D (2020). Coronavirus disease-19 and fertility: viral host entry protein expression in male and female reproductive tissues. Fertil Steril.

[49] Wang S, Qiu Z, Hou Y, Deng X, Xu W, Zheng T (2021). AXL is a candidate receptor for SARS-CoV-2 that promotes infection of pulmonary and bronchial epithelial cells. Cell Res.

[50] Corona G, Vignozzi L, Sforza A, Maggi M (2013). Risks and benefits of late onset hypogonadism treatment: an expert opinion. World J Mens Health.

[51] Salonia A, Pontillo M, Capogrosso P, Gregori S, Carenzi C, Ferrara AM (2021). Testosterone in males with COVID-19: a 7-month cohort study. Andrology.

[52] Corona G, Rastrelli G, Di Pasquale G, Sforza A, Mannucci E, Maggi M (2018). Endogenous testosterone levels and cardiovascular risk: meta-analysis of observational studies. J Sex Med.

[53] Corona G, Rastrelli G, Vignozzi L, Barbonetti A, Sforza A, Mannucci E (2021). The role of testosterone treatment in patients with metabolic disorders. Expert Rev Clin Pharmacol.

[54] Grossmann M, Ng Tang Fui M, Cheung AS (2020). Late-onset hypogonadism: metabolic impact. Andrology.

[55] Coluzzi F, Billeci D, Maggi M, Corona G (2018). Testosterone deficiency in non-cancer opioid-treated patients. J Endocrinol Invest.

[56] Santi D, Spaggiari G, Vena W, Pizzocaro A, Maggi M, Rochira V (2021). The prevalence of hypogonadism and the effectiveness of androgen administration on body composition in HIV-infected men: a meta-analysis. Cells.

[57] Corona G, Goulis DG, Huhtaniemi I, Zitzmann M, Toppari J, Forti G (2020). European Academy of Andrology (EAA) guidelines on investigation, treatment and monitoring of functional hypogonadism in males: endorsing organization: European Society of Endocrinology. Andrology.

[58] Pizzocaro A, Vena W, Condorelli R, Radicioni A, Rastrelli G, Pasquali D (2020). Testosterone treatment in male patients with Klinefelter syndrome: a systematic review and meta-analysis. J Endocrinol Invest.

[59] Sarchielli E, Comeglio P, Filippi S, Cellai I, Guarnieri G, Marzoppi A (2021). Neuroprotective effects of testosterone in the hypothalamus of an animal model of metabolic syndrome. Int J Mol Sci.

[60] Comeglio P, Sarchielli E, Filippi S, Cellai I, Guarnieri G, Morelli A (2021). Treatment potential of LPCN 1144 on liver health and metabolic regulation in a non-genomic, high fat diet induced NASH rabbit model. J Endocrinol Invest.

[61] Corona G, Torres LO, Maggi M (2020). Testosterone therapy: what we have learned from trials. J Sex Med.

[62] Baldassarri M, Picchiotti N, Fava F, Fallerini C, Benetti E, Daga S (2021). Shorter androgen receptor polyQ alleles protect against life-threatening COVID-19 disease in European males. EBioMedicine.

[63] Cheng H, Peng Z, Luo W, Si S, Mo M, Zhou H (2021). Efficacy and safety of COVID-19 vaccines in phase III trials: a meta-analysis. Vaccines (Basel)..

[64] Barda N, Dagan N, Ben-Shlomo Y, Kepten E, Waxman J, Ohana R (2021). Safety of the BNT162b2 mRNA Covid-19 vaccine in a Nationwide setting. N Engl J Med.

[65] Gupta S, Cantor J, Simon KI, Bento AI, Wing C, Whaley CM (2021). Vaccinations against COVID-19 may have averted up To 140,000 deaths in the United States. Health Aff (Millwood).

