# Supplementary material for: Andrological effects of SARS-Cov-2 infection: a systematic review and meta-analysis
Source: J Endocrinol Invest. 2022 May 9;45(12):2207–19. doi: 10.1007/s40618-022-01801-x (PMC9080963; doi:10.1007/s40618-022-01801-x)
Supplement: Supplementary file 2 — Supplementary file2 (DOCX 17 KB) [file 40618_2022_1801_MOESM2_ESM.docx]

**Supplementary Figure 2.** Funnel plot of male genitalia tract SARS-CoV2 mRNA detection rate
