# Supplementary material for: Andrological effects of SARS-Cov-2 infection: a systematic review and meta-analysis
Source: J Endocrinol Invest. 2022 May 9;45(12):2207–19. doi: 10.1007/s40618-022-01801-x (PMC9080963; doi:10.1007/s40618-022-01801-x)
Supplement: Supplementary file 3 — Supplementary file3 (DOCX 45 KB) [file 40618_2022_1801_MOESM3_ESM.docx]

**Supplementary Figure 3.** Male genitalia tract SARS-CoV2 mRNA detection rate (%) according to timing from diagnosis (days). Panel A whole population, Panel B after the exclusion of those studies evaluating the presence of SARS-CoV2 in testis autopsy
