# Supplementary material for: Andrological effects of SARS-Cov-2 infection: a systematic review and meta-analysis
Source: J Endocrinol Invest. 2022 May 9;45(12):2207–19. doi: 10.1007/s40618-022-01801-x (PMC9080963; doi:10.1007/s40618-022-01801-x)
Supplement: Supplementary file 4 — Supplementary file4 (DOCX 40 KB) [file 40618_2022_1801_MOESM4_ESM.docx]

**Supplementary Figure 4.** Semen parameters in COVID-19 subjects as compared to controls when the only study not considering healthy controls [26] was excluded from the analysis: total sperm count (A), sperm concentration (B), semen volume (C).
