# Supplementary material for: Andrological effects of SARS-Cov-2 infection: a systematic review and meta-analysis
Source: J Endocrinol Invest. 2022 May 9;45(12):2207–19. doi: 10.1007/s40618-022-01801-x (PMC9080963; doi:10.1007/s40618-022-01801-x)
Supplement: Supplementary file 5 — Supplementary file5 (DOCX 43 KB) [file 40618_2022_1801_MOESM5_ESM.docx]

**Supplementary Figure 5.** Semen parameters in COVID-19 subjects as compared to controls after the exclusion of those studies considering subjects with positive oropharyngeal swab [11, 13, 36] from the analysis: total sperm count (A), sperm concentration (B), semen volume (C).
