# Supplementary material for: Andrological effects of SARS-Cov-2 infection: a systematic review and meta-analysis
Source: J Endocrinol Invest. 2022 May 9;45(12):2207–19. doi: 10.1007/s40618-022-01801-x (PMC9080963; doi:10.1007/s40618-022-01801-x)
Supplement: Supplementary file 7 — Supplementary file7 (DOCX 40 KB) [file 40618_2022_1801_MOESM7_ESM.docx]

**Supplementary Figure 7.** Total testosterone in COVID-19 subjects vs controls according to clinical diseases phase (A) or type of control populations (B).
