# Supplementary material for: Andrological effects of SARS-Cov-2 infection: a systematic review and meta-analysis
Source: J Endocrinol Invest. 2022 May 9;45(12):2207–19. doi: 10.1007/s40618-022-01801-x (PMC9080963; doi:10.1007/s40618-022-01801-x)
Supplement: Supplementary file 8 — Supplementary file8 (DOCX 95 KB) [file 40618_2022_1801_MOESM8_ESM.docx]

**Figure 8.** Semen parameters before and after COVID-19 vaccination: Sperm concentration (A), sperm total motility (B), and semen volume (C).
