# Supplementary material for: Andrological effects of SARS-Cov-2 infection: a systematic review and meta-analysis
Source: J Endocrinol Invest. 2022 May 9;45(12):2207–19. doi: 10.1007/s40618-022-01801-x (PMC9080963; doi:10.1007/s40618-022-01801-x)
Supplement: Supplementary file 9 — Supplementary file9 (DOC 86 KB) [file 40618_2022_1801_MOESM9_ESM.doc]

| **Author** | **SARS-CoV2**  **detection rate** | **Sperm outcomes** | | | | | **Hormonal outcomes** | | | | **Vaccination outcomes** |
| --- | --- | --- | --- | --- | --- | --- | --- | --- | --- | --- | --- |
|  |  | **Semen volume (ml)** | **Total sperm count (mln)** | **Sperm concentration (mln/ml)** | **Progressive motility  (%)** | **Sperm**  **morphology** | **FSH (U/L)** | **LH (U/L)** | **TT (nM/L)** | **Low T related risk** |  |
| **Çayan et al, 2020 (10)** |  |  |  |  |  |  | X | X | X |  |  |
| **Guo et al, 2020 (26)** | X |  |  |  |  |  |  |  |  |  |  |
| **Holtmann et al, 2020 (11)** | X |  | X | X | X |  |  |  |  |  |  |
| **Kayaaslan et al, 2020 (12)** | X |  |  |  |  |  |  |  |  |  |  |
| **Li et al, 2020* (13)** | X |  |  | X |  |  |  |  |  |  |  |
| **Li et al, 2020* (8)** | X |  |  |  |  |  |  |  |  |  |  |
| **Ning et al, 2020 (38)** | X |  |  |  |  |  |  |  |  |  |  |
| **Pan et al, 2020 (14)** | X |  |  |  |  |  |  |  |  |  |  |
| **Pavone et al, 2020 (15)** | X |  |  |  |  |  |  |  |  |  |  |
| **Rastrelli et al, 2020 (4)** |  |  |  |  |  |  |  |  | X | X |  |
| **Rawlings et al, 2020 (16)** | X |  |  |  |  |  |  |  |  |  |  |
| **Salciccia et al, 2020 (17)** |  |  |  |  |  |  |  |  | X |  |  |
| **Song et al, 2020 (18)** | X |  |  |  |  |  |  |  |  |  |  |
| **Yang et al, 2020 (19)** | X^§^ |  |  |  |  |  |  |  |  |  |  |
| **Achua et al, 2021 (20)** | X^§^ |  |  |  |  |  |  |  |  |  |  |
| **Burke et al, 2021 (21)** | X |  |  |  |  |  |  |  |  |  |  |
| **Camici et al, 2021 (22)** |  |  |  |  |  |  |  |  | X |  |  |
| **Cinislioglu et al, 2021 (23)** |  |  |  |  |  |  | X | X | X | X |  |
| **Dhindsa et al, 2021 (24)** |  |  |  |  |  |  |  |  | X | X |  |
| **Gacci et al, 2021 (25) ^†^** |  | X | X | X | X | X |  |  |  |  |  |
| **Gonzalez et al. 2021 (39)** |  | X |  | X |  |  |  |  |  |  | X |
| **Kadihasanoglu et al, 2021 (27)** |  |  |  |  |  |  | X | X | X |  |  |
| **Lanser et al, 2021 (28)** |  |  |  |  |  |  |  |  |  | X |  |
| **Ma et al, 2021 (29) **** | X |  |  |  |  |  |  |  |  |  |  |
| **Ma et al, 2021 (29) **** |  |  |  |  |  |  | X | X | X |  |  |
| **Machado et al, 2021 (30)** | X |  |  |  |  |  |  |  |  |  |  |
| **Maleki et al, 2021 (31)** |  | X | X | X | X | X |  |  |  |  |  |
| **Okçelik et al, 2021 (32)** |  |  |  |  |  |  | X | X | X |  |  |
| **Ruan et al, 2021 (33)** | X | X | X | X | X |  |  |  |  |  |  |
| **Safrai et al, 2020 (40)** |  | X |  | X |  |  |  |  |  |  | X |
| **Salonia et al, 2021 (5)** |  |  |  |  |  |  | X | X | X | X |  |
| **Saylam et al, 2021 (34)** | X |  |  |  |  |  |  |  |  |  |  |
| **Sharma et al, 2021 (35)** | X |  |  |  |  |  |  |  |  |  |  |
| **Temiz et al, 2021 (36)** | X | X | X | X | X | X | X | X | X |  |  |
| **Xu et al, 2021 (37)** |  |  |  |  |  |  | X | X | X |  |  |

**Supplementary Table I. Outcome parameters reported per single trial included in the meta-analysis. LH**= luteinizing hormone; **FSH**= follicle-stimulating hormone; **TT**= total testosterone; **HPT**= arterial hypertension; **DM**= diabetes mellitus; ***** different study; ******same study, different cohort; ^§^ autoptic series; **†** hospitalized vs non-hospitalized subjects;
