# Supplementary material for: Andrological effects of SARS-Cov-2 infection: a systematic review and meta-analysis
Source: J Endocrinol Invest. 2022 May 9;45(12):2207–19. doi: 10.1007/s40618-022-01801-x (PMC9080963; doi:10.1007/s40618-022-01801-x)
Supplement: Supplementary file 10 — Supplementary file10 (DOCX 23 KB) [file 40618_2022_1801_MOESM10_ESM.docx]

| **Author** | **Study design** | **Selection bias** | **Data collection** | **Overall rating** |
| --- | --- | --- | --- | --- |
| **Çayan et al, 2020 (10)** | Prospective | Low | Moderate | Moderate |
| **Guo et al, 2020 (26)** | Prospective | Strong | Moderate | Moderate |
| **Holtmann et al, 2020 (11)** | Prospective | Low | Moderate | Moderate |
| **Kayaaslan et al,** **2020 (12)** | Prospective | Moderate | Weak | Low |
| **Li et al, 2020 (13)*** | Prospective | Moderate | Moderate | Moderate |
| **Li et al, 2020 (8)*** | Prospective | Strong | Weak | Low |
| **Ning et al, 2020 (38)** | Retrospective | Moderate | Weak | Low |
| **Pan et al, 2020 (14)** | Prospective | Moderate | Weak | Low |
| **Pavone et al, 2020 (15)** | Prospective | Strong | Weak | Low |
| **Rastrelli et al, 2020 (4)** | Prospective | Moderate | Moderate | Moderate |
| **Rawlings et al, 2020 (16)** | Prospective | Strong | Weak | Low |
| **Salciccia et al, 2020 (17)** | Prospective | Moderate | Moderate | Moderate |
| **Song et al, 2020 (18)** | Prospective | Strong | Weak | Low |
| **Yang et al, 2020 (19)** | Retrospective | Strong | Weak | Low |
| **Achua et al, 2021 (20)** | Retrospective | Strong | Weak | Low |
| **Burke et al, 2021 21)** | Prospective | Moderate | Weak | Low |
| **Camici et al, 2021 (22)** | Retrospective | Moderate | Moderate | Moderate |
| **Cinislioglu et al, 2021 (23)** | Prospective | Weak | Moderate | Moderate |
| **Dhindsa et al, 2021 (24)** | Prospective | Weak | Moderate | Moderate |
| **Gacci et al, 2021 (25)** | Prospective | Weak | Weak | Moderate |
| **Gonzalez et al, 2021 (39)** | Prospective | Moderate | Moderate | Moderate |
| **Kadihasanoglu et al, 2021 (27)** | Prospective | Strong | Weak | Low |
| **Lanser et al, 2021 (28)** | Retrospective | Strong | Moderate | Moderate |
| **Ma et al, 2021 (29)** | Prospective | Moderate | Moderate | Moderate |
| **Machado et al, 2021 (30)** | Prospective | Strong | Weak | Low |
| **Maleki et al, 2021 (31)** | Prospective | Weak | Moderate | Moderate |
| **Okçelik et al, 2021 (32)** | Prospective | Strong | Weak | Low |
| **Ruan et al, 2021 (33)** | Prospective | Moderate | Strong | High |
| **Safrai et al, 2021 (40)** | Retrospective | Strong | Weak | Low |
| **Salonia et al, 2021 (5)** | Prospective | Weak | Moderate | High |
| **Saylam et al, 2021 (34)** | Prospective | Strong | Weak | Low |
| **Sharma et al, 2021 (35)** | Prospective | Strong | Weak | Low |
| **Temiz et al, 2021 (36)** | Prospective | Strong | Moderate | Low |
| **Xu et al, 2021 (37)** | Prospective | Strong | Moderate | Low |

**Supplementary Table II.** Quality assessment of the clinical studies included in the meta-analysis.
